# Mainstream Technologies in Facilities for People With Intellectual Disabilities: Multiple-Methods Study Using the Nonadoption, Abandonment, Scale-Up, Spread, and Sustainability Framework

**DOI:** 10.2196/59360

**Published:** 2024-11-05

**Authors:** Christian Menschik, Christophe Kunze, Gregor Renner, Theresa Etges

**Affiliations:** 1 Care & Technology Lab Furtwangen University Furtwangen Germany; 2 Catholic University of Applied Sciences Freiburg im Breisgau Germany

**Keywords:** intellectual disabilities, mainstream technology, technology adoption, technology implementation, NASSS, digital competencies, facility for people with disabilities, mobile phone

## Abstract

**Background:**

People with intellectual disabilities in residential or outpatient facilities for people with disabilities run the risk of being digitally excluded by not having opportunities for taking advantage of digitalization possibilities.

**Objective:**

We aimed to investigate how disability caregivers and managers describe barriers and facilitating factors to implement and adopt mainstream technology for people with intellectual disabilities in residential or outpatient facilities and how the competencies and capabilities of the caregivers are assessed in the process.

**Methods:**

For this reason, we conducted a multiple-methods study applying the nonadoption, abandonment, scale-up, spread, and sustainability framework.

**Results:**

As a result, we identified barriers and facilitators across the nonadoption, abandonment, scale-up, spread, and sustainability framework domains: (1) condition—people with intellectual disabilities are a diverse group, where the individual condition of the person and, for example, their communication skills were seen as a prerequisite for implementing mainstream technologies; (2) technology—the extent to which mainstream technology fits the individual needs and demands contributed to the implementation process; (3) value proposition—communication was seen as a life area where mainstream technology can add value; (4) adopters—the caregivers needed competencies and capabilities to accompany their care recipients’ technology use; (5) organization—missing legal regulations and lack of personnel resources were described as barriers; (6) wider context—funding opportunities were seen as unclear in disability services as mainstream technologies could not be financed as participation benefits; (7) embedding and adaptation over time—the COVID-19 pandemic forced facilities to become digitalized to some extent.

**Conclusions:**

The disability services investigated were still in need of standardized procedures to promote the digital participation of their residents.

## Introduction

### Background

The use of digital mainstream technologies such as smartphones, tablets, or PCs is ubiquitous nowadays and can promote the participation of people with intellectual disabilities. Mainstream technologies have been shown to promote digital participation by enabling people with intellectual disabilities to access digital content (eg, by using voice interfaces) [1]. The use of social media can promote the social participation of people with intellectual disabilities [2,3]. Furthermore, mainstream technologies can even be used as assistive devices, for example, to help people with intellectual disabilities perform daily tasks such as obtaining directions using navigation apps [1,4]. However, people with intellectual disabilities are less likely to use digital technologies [5]. Compared to people with physical or sensory disabilities, the mainstream technology use of people with intellectual disabilities is even more infrequent [6]. Therefore, they run the risk of being excluded from digital possibilities and affected by the digital divide [7]. People with intellectual disabilities often live in residential or outpatient facilities for people with disabilities, which in Germany rarely offer a digital infrastructure for their residents, such as access to Wi-Fi or digital devices [8]. Service-related IT use by disability professionals in Germany, such as digital documentation systems, has increased in recent years [9]. Nevertheless, facilities for people with disabilities in Germany are in a particularly poor position. In their study, Heitplatz and Sube [10] point out that particularly individuals living in residential settings are less likely to have their own internet-ready devices and internet access. This imbalance is also evident when they are compared with people without disabilities. For instance, Adrian et al [11] found that the technological equipment of people with intellectual disabilities in residential care facilities is worse than that in private households of people without disabilities. The effects of the COVID-19 pandemic have internationally brought the digital participation of people with disabilities into the spotlight, especially in residential facilities. Although the pandemic situation pushed digital participation to some extent, it did not ensure that digital exclusion will be overcome [12]. In Germany in particular, the poor digital infrastructure of the facilities and missing support structures have become apparent. The residents’ lack of digital devices, insufficient access to the internet (eg, via Wi-Fi), and a lack of financial resources were revealed [13-16]. Where access to digital technologies is possible, their satisfactory use cannot always be guaranteed. A lack of skills and support in using the technologies and their lack of accessibility have made their use by people with intellectual disabilities difficult [17,18].

On the other hand, there are also reports of positive impacts on the digital participation of people with disabilities. People with intellectual disabilities’ technology use increased during the COVID-19 pandemic. Some people even came into first contact with information and communications technologies (ICTs) due to removing barriers to access by previously gatekeeping caregivers [18-20].

Research indicates that services for people with disabilities had to find inventive strategies to offer digital solutions during the COVID-19 pandemic [12]. However, there is little evidence in the literature on barriers and facilitating conditions under which technologies can be implemented in residential or outpatient facilities for people with intellectual disabilities and how sustainable this can be. Clifford Simplican et al [21] investigated staff members’ perceptions of challenges in integrating new technologies for people with intellectual disabilities in residential settings and found that the challenges involve an interplay of several factors, such as the personal conditions of the residents (eg, age, abilities, or financial resources), skills and time resources of the staff, or ethical and safety issues. Ramsten et al [22] point out that the organizational provision of information about ICT and the development of the staff’s ICT knowledge can be beneficial for developing ICT implementation strategies. However, unclear regulations about policy and funding of digital technologies can be a barrier for people with intellectual disabilities to accessing them [23]. In addition, the implementation of ICT in disability services must fit the daily routines and organizational culture [24]. Therefore, an evaluation of the implementation process by external experts can be seen as problematic due to the missing view from inside the organization [24].

Barriers to and facilitators of implementing new technology are already well explored in other social services such as health care. The nonadoption, abandonment, scale-up, spread, and sustainability (NASSS) framework [25,26] is used to investigate technology innovations in health care organizations such as video consulting systems [27], web-based psychiatric therapy applications [28], or artificial intelligence applications [29]. Therefore, we decided to apply the NASSS framework to investigate mainstream technology adoption in services and supports for people with intellectual disabilities. The NASSS framework originally comes from the objective of examining technology innovation implementation in health and social care institutions through an “evidence-based, theory-informed and pragmatic framework” [25]. According to the NASSS framework, the more complexity there is in a technology-related change process, the less likely the technology is to be implemented sustainably. The framework consists of 7 domains (and subdomains) that help reveal different kinds of complexity: condition, technology, value proposition, adopters, organization, wider system, and embedding and adaptation over time.

### Goal of This Study

To date, there is no research that applies the NASSS framework to investigate mainstream technology implementation in facilities for people with intellectual disabilities. However, we believe that the framework can be useful for this purpose. First, the authors [25,26] point out that the NASSS framework can be applied to investigate health and social care organizations, which includes services for people with intellectual disabilities. Furthermore, we assume that services for people with disabilities and health services may be related as some of them involve similar objectives, such as independent living or self-determined mobility. Greenhalgh et al [30] demonstrate this in their case studies about GPS tracking, pendant alerts, and care organizing software that informed the empirical validation of the NASSS framework. Finally, studies show that the private use of mainstream technologies by people with intellectual disabilities is strongly embedded in and dependent on the structures of the organization they reside in. The residents’ technology and internet access can be dependent on the facilities’ digital infrastructure [6] or on caregivers’ competencies, motivation, and attitude toward technology [31,32]. Therefore, we assume that the use of mainstream technologies by people with intellectual disabilities living in residential or outpatient facilities requires a complex implementation and adoption process that can be analyzed using the NASSS framework. Therefore, we will address the following research questions:

How do disability caregivers and managers describe barriers and facilitating factors to implement and adopt mainstream technology for people with intellectual disabilities in residential or outpatient facilities?

How do disability caregivers assess their capabilities in the technology implementation and adoption procedure?

## Methods

### Design

We used a multiple-methods approach conducting 14 qualitative semistructured interviews with staff of facilities for people with intellectual disabilities about barriers to and facilitators of mainstream technology implementation. As a lack of digital competencies of caregivers is a common barrier to implementation, we additionally conducted a quantitative web-based survey asking 65 caregivers about their basic ICT skill beliefs. The statistical data were meant to complement the qualitative findings in NASSS domain 4 (adopters).

### Interview Study

#### Recruitment

In the qualitative part of the study, we conducted 14 semistructured interviews with managers (n=6, 43%) and caregivers (n=8, 57%) from different residential or outpatient facilities for people with intellectual disabilities in Germany. A total of 57% (8/14) female and 43% (6/14) male persons were interviewed, as shown in Table 1. All caregivers had previous experience in implementing mainstream technology for at least one adult resident they cared for. All managers ran an organization in which mainstream technology was implemented for at least one resident.

**Table 1:** Interview sample.

Pseudonym	Profession	Sector	Gender
ID1	Manager	Residential	Male
ID2	Manager	Residential and outpatient	Male
ID3	Manager	Residential and outpatient	Male
ID4	Manager	Outpatient	Female
ID5	Caregiver	Outpatient	Female
ID6	Caregiver	Outpatient	Female
ID7	Caregiver	Outpatient	Female
ID8	Caregiver	Residential	Female
ID9	Manager	Outpatient	Female
ID10	Caregiver	Residential	Male
ID11	Manager	Residential	Female
ID12	Caregiver	Residential	Male
ID13	Caregiver	Residential	Male
ID14	Caregiver	Residential	Female

#### Data Collection

The semistructured interviews were held in German via a videoconferencing tool and lasted between 20 and 45 minutes. This form of semistructured interviewing allows for a previously defined set of questions to be addressed and also ensures that enough openness is maintained to gain deeper individual insights into the phenomenon of interest by asking how and why questions [33]. The interviews were audio recorded and transcribed verbatim in German using a semantic content transcription system [34].

#### Data Analysis

For analyzing interview data, we applied qualitative content analysis using inductive and deductive coding [35]. Inductive coding was conducted using the MAXQDA software (VERBI GmbH), resulting in an inductive category system that consist of main categories and subcategories. In addition, the interview statements that referred to a certain life area where technology use was described as useful were coded deductively using the domains of activities and participation according to the International Classification of Functioning, Disability, and Health (ICF) [36]. The domains of activities and participation are (1) learning and applying knowledge, (2) general tasks and demands, (3) communication, (4) mobility, (5) self-care, (6) domestic life, (7) interpersonal interactions and relationships, (8) major life areas, and (9) community and social and civic life [36]. This is seen as a framework that covers “the full range of life areas (from basic learning or watching to composite areas such as interpersonal interactions or employment)” [36]. This was used to complement the inductive category system. Subsequently, the NASSS domains were used as a deductive coding system that was applied to the inductive category system and guided the matching between NASSS domains and the inductive category system. Anchor quotes were then translated into English for the publication.

### Survey Study

#### Sample

A total of 65 completed questionnaires were used for the analysis. In total, 65% (42/65) female, 34% (22/65) male, and 2% (1/65) diverse individuals working as caregivers in different residential or outpatient facilities for people with intellectual disabilities in Germany were surveyed about their self-assessment of digital competencies.

#### Data Collection

There are no validated questionnaires for assessing the digital and media competencies of disability caregivers. Therefore, we decided to adapt the self-assessment questionnaire for teachers’ basic ICT competence beliefs by Rubach and Lazarides [37]. The instrument includes the following competency areas: (1) information and data literacy, (2) communication and collaboration, (3) digital content creation, (4) safety and security, (5) problem-solving, and (6) analyzing and reflecting. We used the items for the self-assessment of general, profession-independent competencies in using digital media that apply also to nonschool areas and added a specific competency and capability domain—disability and technology—for adaptation to the services and supports for people with disabilities. The questionnaire was created using the web-based survey tool LimeSurvey (LimeSurvey GmbH) in German.

#### Data Analysis

A total of 65 completed questionnaires were exported from LimeSurvey. Descriptive statistics were used to analyze the data using the Microsoft Excel software (Microsoft Corp). In addition, diagrams that display the findings were created and translated into English. Statistical findings were subsequently included in the analysis of NASSS domain 4 (adopters). The statistical data were used to complement qualitative findings only in this specific domain.

### Ethical Considerations

We obtained informed consent from all participants. We declared that participation in this study was voluntary, that respondents would not be disadvantaged by participating, and that results would be published in a form that would not allow any conclusions to be drawn about the individuals. Participants did not receive financial compensation. This study received a positive ethics approval from the German Society for Educational Science (05/2019/DGfE).

## Results

### Overview

Multimedia Appendix 1 shows the overall category system by NASSS domain and the matching inductive category codes from the interviews, as well as the self-assessed competency and capability areas. The results are described in detail in the following sections.

### NASSS Domain 1: The Condition

The interviewed caregivers and managers indicated that certain individual preconditions of people with intellectual disabilities are advantageous or disadvantageous for the use of digital technologies. Thus, when it comes to the suitability [25] of technologies for people with intellectual disabilities, it is essential to consider each case individually, taking into account their unique personal, environmental, and health factors. Therefore, the ability to verbally express the desire to use technology was described as an important precondition for its implementation. These indications about individual preconditions regard varying kinds of impairments as well as differences in age and previous experience with technologies that condition differing needs and demands:

Well, I would say, ... the group of people living here is very diverse and it always requires individual adaptation. That makes the whole thing a challenge, you cannot say, we simply build ten devices or twenty and distribute them among the residents, so it will not work this way. ... On the one hand there are the physical difficulties, ..., that a person has. ... And the other is/are so the, ... cognitive competences in the end also.

ID1; position 263-269

Referring to the understanding of disability according to a biopsychosocial approach [36], the conditions of the persons concerned vary individually depending on personal and environmental factors as well as their health condition. This impression was also conveyed in the interviews.

In this respect, the interviewees reported, for example, that people with intellectual disabilities can have difficulties with fine motor tasks, which can make the touch operation of a smartphone more difficult. However, speech interfaces were not seen as adequate compensation. The residents’ pronunciation may be slurred, which makes operation via voice control more difficult. Therefore, a satisfactory use was mentioned as a basic requirement for sustaining motivation. However, this can be a matter of previous experience or training. This also applies to the learning of digital and media skills to learn the adequate use of digital technologies:

So it’s like with children, too, you just have to dose it, too, depending. Sure, so if they then only lose themselves in some tablet or mobile games and can no longer really participate in everyday life or the structure in everyday life is lost, because they are only on the tablet or the tablet or cell phone all the time, then of course you have to dose the everyday life.

ID11; position 200-205

According to the statements of the interviewed persons, the older adult residents they care for have less previous experience with technology than the younger residents.

### NASSS Domain 2: The Technologies

To ensure successful technology adoption, the material and technical features of the technologies should be highly linked to the unique conditions of the residents. Therefore, the accessibility of certain technologies plays a primary role. The interviewees referred to the control and customization options of the device or application that need to fit with the needs and competencies of the residents. This was described as challenging due to “a wide spectrum from cognitive impairment, I mean the severity of the disability, to also the severity of motor impairments” (ID1; position 273-276). Thus, as the interfaces of digital technologies usually offer different input modalities, such as touch, speech, or even external switch, the interviewed persons also saw these benefits for certain applications, such as those for messaging:

That’s also very different, so what everyone actually likes to use is WhatsApp, to simply be in the group chat, or even to write people. Of course, the use of voice recording is also very useful, because not everyone can read and write so well.

ID9; position 81-85

The customization options of the device or application were described as helpful. The interviewed employees mentioned, for example, screen enlargements as an easy and quick accessibility tool. Other customization options focused on security and protection measures, including parental controls, ad blockers, and third-party charge blocking, to allow residents to use the system more independently. As mentioned previously, a satisfying technology use may be a matter of previous experience or training. Accordingly, it was described as beneficial if the person is given the opportunity to try things out individually:

So I just installed games for him, this person and I said just try out and then he just worked his way through it and then told me at the end I’ll play these and these games, but I can’t manage the rest. He was able to report back to me, then I chucked the games back down and had a look at what he had mastered and what he was able to do. I then followed that up and downloaded more. Yes. So just tried it out. Yes. And so I would also say, you can’t predict that, you have to try out you have to try out what the person can manage to intellectually cope with.

ID14; position 150-159

A given space for exercising with digital devices may additionally affect issues of sustainability. Thus, when a resident has tried different things, it is easier for caregivers, relatives, or legal guardians to decide what makes sense to purchase in each individual case. The legal guardian then plays a significant role if the residents need support when it comes to the purchase of a device or the conclusion of a contract. However, technical problems can occur regularly regardless of whether residents are able to use the device easily. Continuous accompaniment of the use was described as elementary by the interviewed persons. Therefore, a competent caregiver (see domain 4) who is able to react spontaneously to technical problems was described as important. It is also possible to receive support via the IT management of the facility, even though this was pointed out to be cumbersome.

### NASSS Domain 3: The Value Proposition

This domain addresses supply- as well as demand-side value [25]. In the case of our research, there is not much interest in the supply-side value because with already existing, well-distributed mainstream technology, the business case for these technologies can be neglected. The demand-side value is far more important, emphasizing the need for cost-effectiveness, desirability, safety, and efficacy [25]. Therefore, cost-effectiveness refers to the end-users’ financial resources, which can be scarce among people with intellectual disabilities, as the interviewed staff reported. How desirable a technology is depends, on the one hand, on the person with disabilities and their wishes and ideas. The caregivers’ ability to recognize the needs of people with complex communication problems was described as a challenge when it comes to identifying a certain technology. People with intellectual disabilities who struggle with verbal communication seem to have less access to digital technologies:

So actually our professionalism is already to recognize what someone wants to tell me without being able to put it into words. So register, the need ... works ... well already. Well, it is sometimes a bit tricky.

ID2; position 173-177

The desirability of a technology, on the other hand, is dependent on the balance between risks and potentials, which can be discussed and negotiated among caregivers, relatives, or legal guardians. The interviewed caregivers referred to risks when using digital technologies, such as reducing social interaction, privacy issues, addiction, financial risks and fraud, or an adequate use of certain applications such as those for messaging. To counteract these risks, not only the acquisition of digital and media skills but also the individual presets of the device were seen as crucial, as well as accompaniment of the use by caregivers. For the interviewed persons, the potentials of digital technologies for people with intellectual disabilities weighed much more than the risks. Fundamentally, it is about becoming “a part of society, which is also appreciated in this area” (ID2; position 343). This became especially significant during the COVID-19 pandemic, when many areas of life shifted to digital platforms and the participation in social interaction required access to videoconferencing tools. Thus, desirability in this case can be discussed concerning the participation rights of people with intellectual disabilities. Therefore, efficacy is analyzed by asking how the technology contributes to participation. When it comes to the efficacy of digital technologies for people with intellectual disabilities, the interviewees referred to certain life areas according to the ICF [36] where digital technologies for promoting participation seem suitable. Communication is the life area that was mentioned the most by the interviewees as that in which some residents already use digital technologies and that holds the biggest potential for participation:

Two of the three residents with down syndrome in our group, they have a cell phone and of course they can and of course they have the possibility to call home all the time. That is fortunately possible nowadays, even if the people, or now in particular the people I know people I know don’t know how to use a smartphone, there is the possibility of using the possibility of calling someone with a simplified cell phone. And that is also frequently used.

ID13; position 62-68

Other life areas mentioned that seemed to be relevant for the participation of residents were community and social and civic life, specifically recreation and leisure activities such as gaming, listening to music, and watching videos, as well as the mobility domain. Traveling alone and independently on public transportation can be a big issue for people with intellectual disabilities. This was stated by the interviewees related to using bus or train schedule apps, using the phone in an emergency, or even using GPS trackers to surveille the location of a resident. Appropriate mobility can open up opportunities such as employment-related matters:

We have actually thought about that at some point he ... attends a vocational preparation facility no sheltered workshop, but rather tries to gain a foothold in the first labor market. But then he has to travel from [place] to [place], 20 minutes by train, but I think we can trust him to do that. And if he then simply has the security that he can reach various people with his smartphone to reach different people relatively unproblematically if the track suddenly suddenly the track changes or something else, is good, I think.

ID9; position 311-320

There were less frequent mentions of the areas of learning and applying knowledge, general tasks and demands, domestic life, and interpersonal interactions and relationships. There were hardly any statements in the areas of self-care and major life areas.

### NASSS Domain 4: The Adopter System

This domain focuses on the practices, roles, and identities of staff members and caregivers, as well as the technology adoption by the patients or residents [25].

#### Residents

With respect to acceptance and adoption of technology by the residents, the interviewees reported on the actual technology use and successful technology adoption by some residents. As mentioned previously, it was stated that people with disabilities who are living in outpatient facilities are more likely to use digital technologies. Therefore, individual adaptation and customization of the devices can be beneficial. Moreover, it was reported that the wish to use technology can also originate from symbolic meanings and esthetics. The need to participate in a digitalized society can be based on the residents’ idea of mainstream technologies as a “status symbol” (ID2; position 111). Again, the condition of the resident was seen as a prerequisite for whether they had their own device and the extent to which independent use was imaginable:

So it always depends on the degree of impairment. Of course, the more they need support, the more difficult it is for them to have their own device or to have it permanently in their room or whatever. You just have to use it in small doses in everyday life, where it would be practical.

ID11; position 82-86

With respect to the financial background of the residents, the interviewees reported on the precarious financial situations of some residents, which are more likely to affect people in outpatient assisted living facilities than in residential care. This may have an impact on the adoption of a new technology:

Then I hope that ... either technology is so cheap to get and also the maintenance of this technology, that people on social welfare level can afford proper technology, without no longer being able to afford bread. Because that is ... a huge issue in my opinion, also a huge issue why technology may not arrive at some ... especially at the people with assistance needs. Of course we also have people where money is in the family background and who then has an Apple and an iPhone and whatever. But THEY do not have the problem. It is now about describing the problem. The problem is that there are some people in our facility who have to make sure that they can afford the co-payment for the medication. ... And if I then say, Yes, a larger tablet would be better for you, then it becomes difficult, doesn’t it?

ID3; position 345-357

With respect to the background of the residents, the interviewed persons also mentioned different family backgrounds that can affect the adoption of a new technology:

Sometimes it was not easy to convince the legal guardians that it is not bad. But that it is actually only about benefiting someone.

ID3; position 386-388

Therefore, family members or legal guardians and their attitude toward technology play an important role in providing individuals with access to certain technologies.

#### Staff and Caregivers

In our study, there were few data concerning staff-related issues. The interviewed managers referred to these issues as a matter of motivation that is linked to their private technology use. Thus, their technology acceptance and attitude influences to what extend technology use is enabled for the residents. They somewhat function as gatekeepers for digital participation:

So ... just as I reach limits in my private life, the users here, the clients also reach limits that I would like to have that it is at least not worse than at my home.

ID2; position 78-80

As the interviewees all worked in facilities that were run by a welfare association, it was reported that the association’s attitude toward technology must be kept in mind:

There are also fears in the association, but also among the employees. The feeling of being under surveillance. We have surveillance fantasies, surveillance fears. Personally, I don’t have it that much.

ID1; position 245-248

Caregivers play a central role in sustainable technology adoption for several reasons. First, the interviewed persons stated that caregivers and their private technology use act as role models for the residents. Individuals who live in residential homes seem to lack social interactions outside the residential setting, and therefore, “people who live here also want to be like the others and in this case company is mainly the staff” (ID2; position 154-156). In this sense, it stands to reason that interviewees stated that caregivers’ attitudes toward technology influence the extent to which residents are enabled to use technology:

So if I’m working in a team where all the employees already don’t enjoy using their devices or leave their cell phones at home and prefer to have nothing to do with technology, then the opportunities for residents are smaller.

ID2; position 216-219

Personal motivation and attitude toward technology can depend on age and, therefore, technology normalization, as stated by the interviewed staff. This then has an impact on whether a mandate is established to promote the digital participation of the residents:

I also see it in older colleagues, not only older colleagues, but for the most part. There are also older colleagues who are very reflective and are very busy with giving people participation and self-determination and yes, but they [laughs] are disabled people he or she doesn’t need that, he or she doesn’t need that, because he or she is disabled. I think that is still difficult to understand.

ID13; position 208-214

When accompanying technology use in practice, caregivers must first assess which technology with which customization or adaptation makes sense in which area of life. Digital and media competencies seem to be a beneficial precondition for that.

#### Digital Competencies of Caregivers

**Figure 1:**
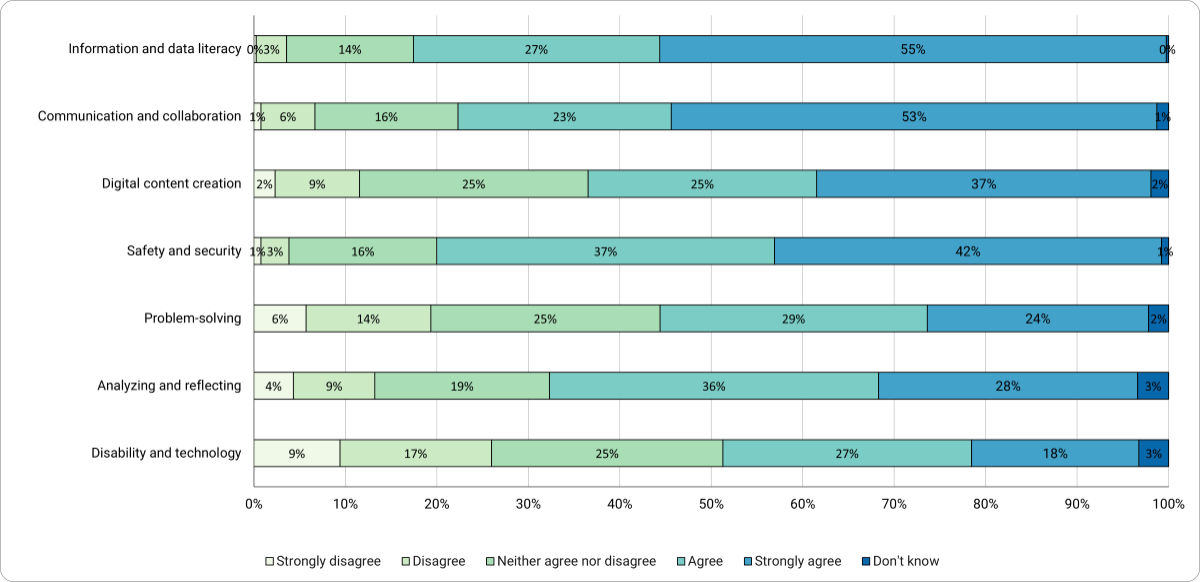
Caregivers’ basic information and communications technology competence beliefs.

In this section, the results of the self-assessment survey of basic ICT competence beliefs of caregivers in disability care are presented (Figure 1).

The survey about competence beliefs in the area of communication and collaboration revealed that 53% (34/65) of the participants strongly agreed. In this area, the associated items deal with communication via digital media such as Skype, quoting and passing on information, jointly editing files and documents, rules of conduct when communicating in the digital space, active participation in society through the use of digital media, and passing on one’s own media experiences to others (eg, recommending and explaining apps).

As reported in the interview study, safety concerns play an important role in relation to the adequacy of content and frequency of use. In the survey study, the area of safety and security showed strong agreement from a large percentage of respondents (27/65, 42%). There seemed to be uncertainties about one’s own skills in the area of analyzing and reflecting (18/65, 28% strongly agreed). The items in this area represent the ability to evaluate the impact of media in the digital space and the ability to evaluate content, such as advertising and fake news, as well as the benefits and risks of business activities on the internet.

Skills such as using and adapting digital tools, organizing learning resources, developing one’s own technical solutions, and recognizing algorithmic structures seemed to be less present in the group of respondents. These skills represent the area of problem-solving, which achieved a low assessment with full agreement in the overall sample (16/65, 24%).

**Figure 2:**
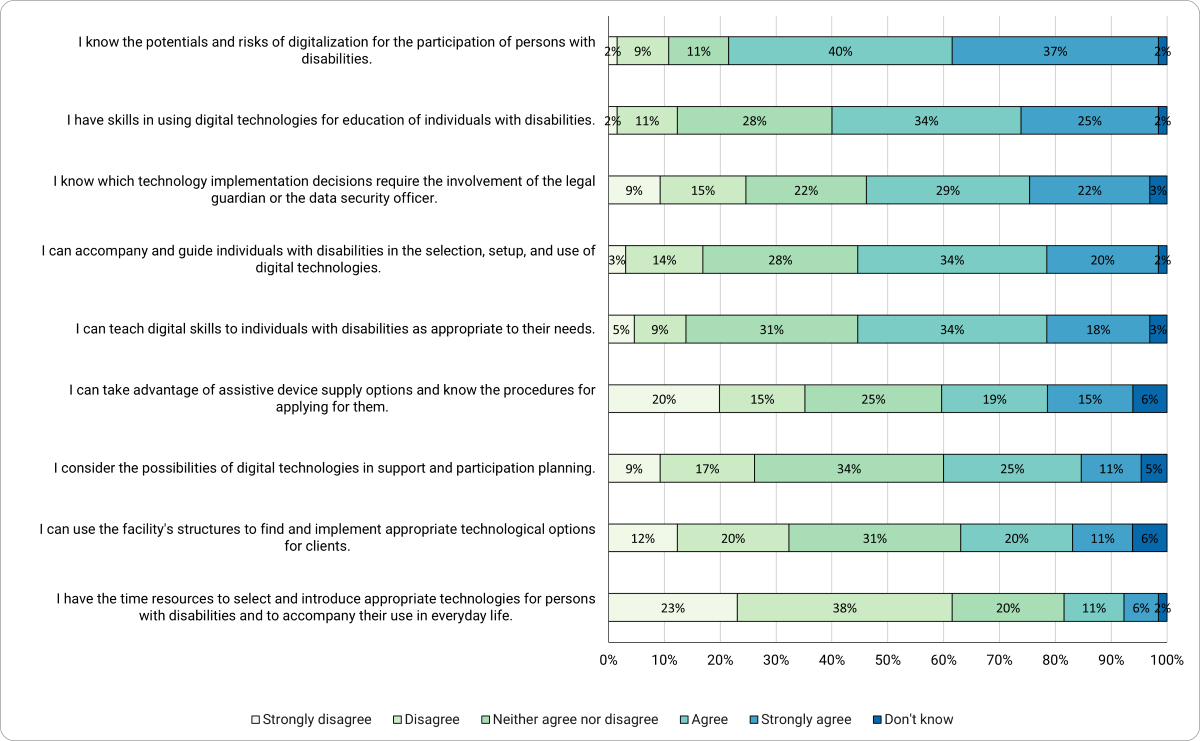
Caregivers’ competence beliefs in the area of disability and technology.

The area of disability and technology (Figure 2) that was added to the original survey showed the lowest level of strong agreement (12/65, 18%) compared with the other areas. For this reason, and because this was considered the most relevant area of self-assessment for our study, the corresponding items are presented in detail, whereas the data of Figure 1 are summaries of different items that are not presented in detail.

The strongest agreement was found with the item of risks and potentials. Taken together, 77% (50/65) of the respondents strongly agreed or agreed that they knew the potentials and risks regarding the digital participation of persons with disabilities.

When asked about the ability to include technologies in educational work, 25% (16/65) of the respondents considered themselves to be fully capable of using digital technologies for pedagogical activities, whereas 34% (22/65) would say that they were to do so. In total, 40% (26/65) of the respondents would only be partially or rather not confident to do this.

With respect to consulting data security officers or legal guardians for certain decisions, 51% (33/65) of the surveyed employees indicated awareness by responding with agreement or strong agreement.

A total of 54% (35/65) of the surveyed staff members in disability care saw themselves as fully or rather competent in guiding and selecting digital technologies and accompanying technology use for people with disabilities, and 28% (18/65) seemed to be partially confident in this.

When asked about media pedagogic skills, 18% (12/65) of the respondents saw themselves as fully capable and 34% (22/65) saw themselves as rather capable of teaching digital skills to the people with intellectual disabilities they cared for.

Employees seemed to be uncertain about the provision of assistive devices. Only 34% (22/65) of the respondents knew the possibilities and procedures for applying for assistive devices. In total, 25% (16/65) seemed to have partial knowledge in this area, whereas 35% (23/65) disagreed or strongly disagreed. Thus, this proportion was higher than that of people who felt rather confident in these skills.

Only 11% (7/65) strongly agreed with the question of considering the possibilities of digital technologies in support and participation planning. Another 25% (16/65) simply agreed, and 26% (17/65) appear to take no account of this aspect in participation planning.

The last 2 items of the disability and technology area of the survey address caregivers’ capabilities to use the structures of the organization and, therefore, are presented in the following section.

### NASSS Domain 5: The Organization

This domain addresses issues related to the organization’s capacity and readiness (or, rather, willingness) to adopt certain technologies [25]. In our interview study, we found statements by the employees about human and other resources committed to digitalization issues. It was described as advantageous to assign additional professionalized staff for ICT and digital issues. However, people coming from a different professional background may have reservations about providing access to the internet for people with intellectual disabilities:

Exactly, we have our system administrator at [the institution], of course, who is responsible for all questions relating to PCs and Wi-Fi and so on. As I said, he was also against setting up a Wi-Fi via [the institution] for a long time. Simply because of liability reasons.

ID9; position 254-257

When asked about the organization’s readiness to implement digital technologies in the residents’ everyday lives, first, the digital infrastructure can be focused. We found that the interviewees reported on a well-used digital infrastructure and a wide use of service-related technologies by the caregivers, such as digital documentation systems or digital timetables. This does not apply to the private technology use of the residents. Thus, a digital working sphere is not yet a prerequisite for a digital private environment for the residents. In the case of the latter, the interviewed persons stated that they found individual solutions to set up a digital infrastructure. There was no standardized procedure mentioned here. Instead, they reported that it must always be examined on an individual basis (eg, who uses the internet to what extent and who pays for it and how). Thus, this depends on the type of accommodation and whether the individuals live in residential group homes that come close to a form of nursing home or in outpatient facilities where they live in a shared flat or on their own with partial support from caregivers. However, with regard to the facility-wide Wi-Fi equipment, various circumstances were mentioned that seemed unclear. On the one hand, interviewees referred to the costs and the issue of whether, for example, the residents themselves should or could pay for the Wi-Fi access or whether a fund could also be considered. In addition, the shared use of the network by residents and caregivers could cause difficulties, especially with regard to data protection issues. It was also not clarified how the costs of internet access could be divided up if the Wi-Fi is used to different extents by different residents. There appears to be a difference between residential and outpatient facilities:

It’s trickier to do this than it is now in a shared apartment where we simply provide outpatient support. Of course, the residents can purchase the Wi-Fi together, which is available on site, and depending on whether they live on the edge of the forest or somehow in the middle of it, it works more or less well, and then it’s more of an agreement among the residents of this shared apartment what they want to spend, what they need. It will always be about fairness somehow. But that’s just their topic, you can support them and here in the facility it’s just rather my topic, so (4) which concerns well, fairness and all the things ....

ID2; position 290-299

The scarce time resources of the employees were stated as crucial and must be kept in mind when it comes to (additional) work and routines that are involved in the technology implementation process. This depends on the individual cases and the residents’ and caregivers’ needs and competencies, as mentioned previously. In our survey study, we found that the item with the lowest level of strong agreement was the issue of time resources. Respondents were asked about the time resources they had for selecting technology and for introducing and accompanying the use of technology in everyday life. A total of 62% (40/65) of the respondents stated that they had no time or rather no time resources for this. Another 20% (13/65) had only partial time resources for this.

Finally, the interviewees reported that facilities face an external pressure to provide the residents they host with an environment in which they can easily use their digital devices. This concerns, for example, the issue of whether the institution provides offers and information for residents and caregivers on the use of digital technologies:

I would say over half have a smartphone definitely and use that. Yes. Exactly so I think that’s coming. The facilities are exposed to this pressure, that there are also the respective needs there and that then also appropriate offers are made. That will also be the case with us.

ID14; position 300-304

In our survey study, we found that only 11% (7/65) of the respondents strongly agreed that they could use the facility’s structures to find and implement suitable technological options for their clients. A total of 20% (13/65) of the respondents somewhat agreed.

### NASSS Domain 6: The Wider Context

The wider context in the case of our study relates to legal issues. On the one hand, there is social legislation and the Bundesteilhabegesetz (a federal participation act in Germany that oversees integration aid in accordance with the Convention on the Rights of Persons with Disabilities) as the basis for legal options for action. As mentioned previously, the issue of whether digital technologies and internet access must be considered as a participation benefit and, therefore, included in assistance plans was brought up. In contrast, the interviewees reported that there are individual issues, such as giving consent to data protection regulations, conclusion of contracts, or the like, where legal guardians also become relevant.

When addressing the adoption of a new technology, one might assume that the technology use of the residents is a private matter and depends on the individual financial resources. However, the interviewees reported that it would be beneficial if digital mainstream technologies were understood as participation benefits so that they could be funded via public health care like other assistive devices. For the interviewees, another possibility seemed to be funding options provided by nongovernmental organizations such as Aktion Mensch (a German nongovernmental organization addressing participation and inclusion issues). Participant ID9 (position 86-93) reported, for example, that training PCs were funded via Aktion Mensch.

### NASSS Domain 7: Embedding and Adaptation Over Time

As has become clear, the individual domains can only be separated from each other analytically as many things overlap or repeat in different contexts. It also has become clear that there are missing strategies in facilities for people with intellectual disabilities, which makes it difficult to promote technology use in a sustainable way. There were statements by the interviewed staff members about how technology should be used in the long term. Mainstream technologies, for which there is hardly any support provided by the supplier, can become buggy over time and, therefore, residents need competencies to deal with such things:

That is finally we do, we do, we do and then it is lying around at some point and no one can take care of it.

ID3; position 425-426

The fact that there are no standardized strategies in the organization also means that technology implementation is an individual process where there is an idea or a need at the beginning that needs to be investigated in terms of suitable technology use. This can be challenging and a matter of “perseverance” (ID1; position 350):

I have experienced too many things in my professional years, where I myself also and yes, or I have only seen observing, there are great ideas on the desk, but then actually fails implementing them.

ID1; position 355-358

In terms of organizations’ innovation process, interviewed staff members reported that the COVID-19 pandemic had a noticeable impact on service-related digital infrastructure as well as residents’ opportunities to access the internet and digital technologies. Setting up videoconferencing systems for staff and residents seems to be the most widespread effect of the pandemic. Enabling residents to communicate with relatives was described as elementary during the pandemic, although this also revealed problems:

So not only we have to overcome the barrier, but also the relatives. We have 80-year-old relatives who have a tablet in their hands for the first time and have purchased it, and then it is also difficult until a connection is possible.

ID1; position 293-297

## Discussion

### Principal Findings

In our study, we examined ex-post barriers and facilitating factors for the implementation of mainstream technologies for people with intellectual disabilities in residential and outpatient facilities of disability services applying the NASSS framework. This study showed that successful technology implementation for the target group depends on complexities in different dimensions that influence each other in different ways.

With respect to the condition of the assisted persons, it was shown that the group of assisted persons consists of highly individual cases with very unique and diverse prerequisites. In the field of disability care, condition can be described as disability concerning the interplay between body functions and structures, activities, and participation, as well as environmental factors according to the ICF [36]. Within the NASSS framework, the condition dimension describes the health situation of a person, including comorbidities and sociocultural aspects. We slightly adapted the operationalization of this dimension accordingly to suit the specifics of the disability perspective. In this case, the special disability-related needs and demands, as well as individual competencies, experiences, and life circumstances, play a role. It was shown that, overall, it was seen as a facilitating factor if the person being cared for is able to communicate their wishes and needs with regard to the use of technology and, thus, provides the impetus for technology implementation. Barriers and facilitators in the technology dimension refer to the degree to which the technology is adaptable to the individual needs and competencies of the person being cared for. The interviewees related the value proposition of a newly introduced technology to different areas of life, whereby it was noted that technologies can be used primarily in the area of communication support. This seems to be a paradox as the communication of a desire for technology use is seen as an important prerequisite for its implementation. Caregivers also play a major role as adopters, on the one hand as they have to interpret needs regarding a wish to use technology in persons with complex communication problems, as the interviews revealed. On the other hand, the survey study showed that the digital competencies of the caregivers were relatively good but they failed in the application of these competencies in the target group, as well as not having enough time resources available. This is linked to the organization domain, where the interviews showed that there was often a lack of sufficient human resources. In addition, the institutions lacked clear guidelines and strategies for implementing technology, which means that this remains a highly individual, nonstructured process. The wider context is also unclear as the promotion of digital participation is not clarified in detail and the issue of whether mainstream technologies count as participation benefits remains open. Finally, it was reported that the COVID-19 pandemic forced facilities to promote digital participation among residents to some extent. However, it remains unclear how sustainable these changes were.

### Comparison With Prior Work

To shed light on the findings in terms of the existing literature, a review of previous studies shows that caregivers have been forced to provide access to digital technologies that they had denied their care recipients before the COVID-19 pandemic [19]. We further showed that it is important for caregivers to be able to appropriately interpret the needs of the person being cared for if the person cannot communicate the desire to use technology themselves. This would mean that particularly people with complex communicative problems bear an increased risk of being excluded from digital participation, especially by gatekeeping caregivers. This refers also to the access to digital information. The use of digital technologies seems to be a challenge for people with low literacy skills [38]. Our findings indicate that the degree to which technology is adapted to individual conditions is important. This is in line with other research in which individual customization of a new technology was a main facilitator for successful adoption [39]. At the same time, it should not be neglected that individual training is especially important for previous nonusers. In the institutions we studied, there were hardly any offers to promote skills that are important for technology use. Whether experience using technology has already been gained depends on the social environment. Friends, family, and even caregivers also act as role models. Other studies have shown that, for example, a peer-to-peer approach can be fruitful, in which people with disabilities are tutors and support other people in technology use [19,40,41].

As studies have shown [6,10], it is people in residential settings who lack digital participation. A tension arises here that indicates that the form of living has an influence on how easily a technology can be implemented. Although both residential and outpatient forms of care are considered as the private living environment of the persons being cared for, residential settings seem to depend on more complex institutional parameters. In outpatient settings, for example, the installation of Wi-Fi can be based on informal agreements among the residents whereby the internet connection is purchased directly from the provider. However, in residential facilities, the internet connection seems more likely to be considered as a facility-wide issue for which the approval of the IT supervisor is required. Parsons et al [24] have shown that staff members can be critical of the external evaluation of implementation projects. On the basis of our results, we argue that, despite a highly individualized, internal implementation process, there is a need for quality control measures to ensure digital participation. In addition, we have shown that the ability to communicate a desire to use technology is a facilitating prerequisite. It can be assumed that this ability is more likely to apply to people in outpatient settings as they require less support.

It also stands out that caregivers have a significant influence on whether a person in care uses a digital technology. However, as an extension to the work by Heitplatz et al [31], we assume that the introduction of new technologies for the target group does not only depend on the attitude of the caregivers toward the technologies. It also depends on the areas of life in which the technology is to be used and how useful this is considered to be. Supporting communication needs through technology is seen as highly useful. In contrast, self-care and independent living is hardly seen as an area of life in which the technology use of people with intellectual disabilities should be promoted. Thus, we agree with Clifford Simplican et al [21] that the caregivers’ attitude toward technology is related to the attitude toward the client and the assessment of the client’s abilities. In addition, the caregiver acts as a role model with their own technology use. We also showed that the caregivers interviewed rated their competencies in their own use of technology as relatively high but the application of these competencies in the media education setting with the individuals being cared for was largely absent. Thus, in this regard, we would add to the work by Ramsten et al [22] that simply promoting caregivers’ ICT competencies is not enough, and therefore, media pedagogical training is needed to support technology use and promote technology adoption by the people cared for. In addition, there are organizational parameters, especially the lack of personnel and time resources of the caregivers, that can prevent technology implementation. The additional professional roles and tasks need to be backed up with time and human resources but are assumed to have been implicitly fulfilled within existing precarious care structures.

Finally, our results show that there are ambiguities in sociolegal regulations (eg, related to the financing of technologies to promote participation). As Boot et al [23] showed, one of the biggest barriers to people with intellectual disabilities accessing assistive technology is a lack of clear regulations, policy, and funding. We argue that mainstream technology implementation can be accomplished without clear legal regulations as a highly individual, nonstructured process, as the results show. However, there is a need for clear regulations in German disability services, which would bring more clarity to this process. In this sense, Bruland et al [42] also confirm this with regard to financing. They state that public health insurance in Germany only finances the provision of an assistive device if it is not an item of daily use. These are understood to be items that are not specifically designed for people with disabilities. However, as assistive systems appear to be integrated into mainstream technologies such as tablets, such a distinction is becoming increasingly problematic. This is also relevant as the lack of financial resources of people with intellectual disabilities in Germany [14,16] is also reflected in our results.

### Limitations

The limitations of this study concern methodological issues. The experimental and multiple-methods approach means that no generalizable statements can be made. This would require more extensive and standardized studies and larger sample sizes for the qualitative as well as the quantitative parts of the study. In this way, due to more data richness, solely qualitative or quantitative studies could also be carried out. There is a risk of sampling bias as caregivers and facility managers with more experience using digital technologies may have been more motivated to take part in the study. In addition, there is no standardized method or questionnaire that collects the ICT competencies of employees in the disability service. Therefore, we had to adapt an existing questionnaire to the field of disability services.

### Conclusions

As people with intellectual disabilities in residential or outpatient facilities for people with disabilities belong to a group of people who, for various reasons, have unfavorable preconditions for taking advantage of the opportunities offered by digitalization, we explored barriers to and facilitating factors of the implementation of mainstream technologies. For this, we applied the NASSS framework and showed that successful technology implementation is accomplished in different dimensions that interact with each other. According to our findings, practitioners in disability services face the challenge of developing distinct tools to identify residents’ technology needs, developing guidelines that ensure sustainable technology implementation in the facilities, and establishing measures that promote employees’ digital and pedagogical skills to accompany the residents’ technology use. Future research should address the issues of how to promote the digital participation of people with intellectual disabilities and how to guide and accompany the different actors in residential and outpatient settings to make appropriate technologies available to the target group. The focus should be on areas of life in which digital participation has received little attention to date.

## Appendix

Multimedia Appendix 1 Nonadoption, abandonment, scale-up, spread, and sustainability (NASSS) framework code matching.

## Abbreviations

ICF: International Classification of Functioning, Disability, and Health
ICT: information and communications technology
NASSS: nonadoption, abandonment, scale-up, spread, and sustainability
