# Supplementary material for: Mainstream Technologies in Facilities for People With Intellectual Disabilities: Multiple-Methods Study Using the Nonadoption, Abandonment, Scale-Up, Spread, and Sustainability Framework
Source: JMIR Rehabil Assist Technol. 2024 Nov 5;11:e59360. doi: 10.2196/59360 (PMC11576602; doi:10.2196/59360)
Supplement: Multimedia Appendix 1 [file rehab_v11i1e59360_app1.pdf]

| NASSS domain and subdomain | Main categories | Subcategories and subsubcategories |
| --- | --- | --- |
| **Condition** | | |
| • Nature of condition or illness | • Barriers to and facilitators of technology adoption | • The role of people with disabilities (needs, demands, and competencies) |
| • Comorbidities and sociocultural influences | • Barriers to and facilitators of technology adoption | • The role of people with disabilities (previous experience with technology and motivation) |
| **Technology** | | |
| • Material features | • Barriers to and facilitators of technology adoption | • The role of technology (control and customization options and accessibility) |
| • Type of data generated | —[a] | — |
| • Knowledge needed to use | • Barriers to and facilitators of technology adoption | • The role of people with disabilities (competencies)<br>• The role of caregivers (digital and media competencies)<br>• The role of technology (exercise options) |
| • Technology supply model | • Actual technology use | • Selection and purchase of apps and devices, initiative for purchase of apps and devices, and support with technical problems |
| **Value proposition** | | |
| • Supply-side value (to developer) | — | — |
| • Demand-side value (to the patient) | • Barriers to and facilitators of technology adoption | • The role of the domains of activities and participation (ICF[b])<br>• The role of technology (risks and potentials) |

| | | | |
|---|---|---|---|
| | | | • The role of caregivers (ability to recognize the needs of the target group) |
| **Adopters** | | | |
| | • Staff (role and identity) | • Barriers to and facilitators of technology adoption | • The role of the institution (motivation of manager and attitude toward technology) |
| | • Patient (simple vs complex input) | • Barriers to and facilitators of technology adoption | • The role of people with disabilities (needs, and demands, financial resources)<br>• The role of legal representatives<br>• The role of relatives and friends |
| | | • Actual technology use | • Equipment of the residents<br>• Technology use by the residents |
| | • Carers (availability and nature of input) | • Barriers to and facilitators of technology adoption | • The role of caregivers (digital and media competencies, attitude toward technology, accompanying technology use, time resources, ability to recognize the needs of the target group, motivation, mandate to promote participation, and caregiver as role model) |
| **Organization(s)** | | | |
| | • Capacity to innovate (eg, leadership) | • Barriers to and facilitators of technology adoption | • The role of the institution (human resources and motivation of manager) |
| | • Readiness for this technology or change | • Barriers to and facilitators of technology adoption | • The role of the institution (attitude toward technology, digital infrastructure, and conceptions) |
| | • Nature of adoption and funding decision | • Barriers to and facilitators of technology adoption | • The role of the institution (conceptions)<br>• The role of people with disabilities (financial resources) |

| | | | |
|---|---|---|---|
| | • Extent of change needed to routines | • Barriers to and facilitators of technology adoption | • The role of caregivers (time resources and capabilities) |
| | • Work needed to implement change | • Barriers to and facilitators of technology adoption | • The role of the domains of activities and participation (ICF)<br>• The role of caregivers (time resources and capabilities)<br>• The role of the institution (provision of offers and information) |
| **Wider system** | | | |
| | • Political<br>• Legal<br>• Professional<br>• Sociocultural | • Barriers to and facilitators of technology adoption | • The role of the institution (legal conditions) |
| **Embedding and adaptation over time** | | | |
| | • Scope for adaptation over time | • Actual technology use | • Sustainable technology use |
| | • Scope for adaptation over time | • Barriers to and facilitators of technology adoption | • The role of the institution (digital infrastructure, conceptions, and motivation of manager)<br>• The role of caregivers (motivation) |
| | • Organizational resilience | • Impact of the COVID-19 pandemic on digitalization | • At the level of the institution, caregivers, and residents and relatives |

[a]Not applicable.

[b]ICF: International Classification of Functioning, Disability, and Health.
